# Identification of Fibre Content in Edible Flours Using Microwave Dielectric Cell: Concise Review and Experimental Insights

**DOI:** 10.3390/ma15165643

**Published:** 2022-08-17

**Authors:** Ashok Ramasamy, Sundaram Muniyasamy, Robert Čep, Muniyandy Elangovan

**Affiliations:** 1 Department of Electronics and Communication Engineering, Kamaraj College of Engineering and Technology, Virudhunagar 625701, India; 2 Department of Electronics and Communication Engineering, Erode Sengunthar Engineering College, Erode 638057, India; 3 Department of Machining, Assembly and Engineering Metrology, Faculty of Mechanical Engineering, VSB-Technical University of Ostrava, 70800 Ostrava, Czech Republic; 4 Department of R&D, Bond Marine Consultancy, London EC1V 2NX, UK

**Keywords:** attenuation, edible flours, microwaves, substances

## Abstract

The quality of edible intake decides the health of the human body and is also responsible for building a healthy immune system in the body. A healthy immune system can protect the body even from invisible attacks of viral or bacterial infections. The assessment of the quality of edible items is not well defined and standardized in many developing countries due to quality assessment difficulties in practice. An alternative well-defined quality assessment approach for edible flours is presented in this paper. Every edible substance has dielectric properties, and it varies from material to material in nature. Edible flours and liquid have different microwave absorption capabilities, based on their natural molecular structure. Based on the microwave energy absorption characteristics of materials, the attenuation constant of edible flours is derived by the waveguide method in this work. In this approach, microwave energy absorption of the edible samples of different types of wheat, rice and millets are observed, and the attenuation constant factors of the samples are then calculated from the tabulated values. The work focuses on the identification of fibre content present in the edible flours. Inferences are made based on the attenuation and its variations with the number of samples, dielectric loss and dielectric constant of the samples. A systematic and concise review of the topic is also included for the benefit of future researchers.

## 1. Introduction

The human health care industry is trying a lot to save human life by fighting against various diseases. All the ancient traditional medical literature says that lifestyle and food habits are the significant factors to cause disease in the human body and they teach us that prevention is better than cure. To achieve the prevention of disease, one of the best practices is to take healthy foods regularly as our intake. The quality of the food intake should be properly defined and its quality assessment is the most important one in achieving disease-free life. The quality assessment of edible items can also be attained by the application of engineering conceptions in addition to the biochemical tests that are available in practice.

The electrical properties of agricultural materials have been of interest for many years. The interest in the dielectric properties of materials has historically been associated with the design of electrical equipment [1]. The study of the thermal behaviour of these edible products requires the accurate determination of the complex permittivity and permeability when the temperature varies and when the materials are irradiated in specific conditions [2]. The dielectric properties of a material are intrinsic electrical properties that describe its polarization states when subjected to an electric field.

At microwave frequencies, polarization is orientation resulting from the rotation of free water molecules in presence of an electric field or reorientation of bound water molecules in association with the food molecules. The above phenomenon depends upon the composition of the material and is affected by the presence of free water, bound water, surface charges, electrolytes, non-electrolytes and hydrogen bonding in the food product. The dielectric properties of edible flours attenuate the microwave energy, based on the fibre content of edible flours [3]. The dielectric properties of edible flours provide useful information to improve the design, processing, quality and control of the product. Among various techniques, opting for an appropriate method requires the consideration of various factors. The electrical characteristics of every material are different to each other, which are dependent on its dielectric properties [4].

An open-ended coaxial-line probe was used with sample temperature control equipment designed for use with the probe to measure the permittivity of some liquid, semi-solid and pulverized food materials as a function of frequency and temperature [5]. The literature says that the dielectric properties of various agri-foods and biological materials are depending upon the frequency range of the emerging signal and the composition of the material [6,7]. Food electric properties are the measures of the food properties, when exposing food material to an applied electric field. Each food component interacts differently with the electric field, since the electric properties of food materials are influenced by their composition, especially the moisture, sugar, salt and fibre content [8]. Food quality includes external and internal factors, such as physical appearance (shape, colour, gloss and consistency), texture, flavours and grade standards, as well as chemical and microbial content [9].

The dielectric properties (έ and ἔ) of edible fungi were measured and inferences were made about the relationship of dielectric properties to their moisture content, frequency and temperature [10]. The quality characteristics of wheat flour, i.e., total protein, crude fibre and total carbohydrates, were measured using microwave signal with various power. The main objective of this research is to choose such global measures from the analysis of the observations made by the experiments. Thus, the measurement of these properties of edible items through microwave waveguides will be a suitable approach for the quality assessment tasks. In the literature review of this work, even though many research works have been presented in this field for the quality assessment of edible items, focusing on unique global measures has not been taken into account. The lack of finding global measures in the research is addressed in this work to assure a reliable prediction about the quality of edible items. Then, this work will have uniqueness in the field of the quality estimation of food items with minimum time consumption for getting the results and less running expenses for completing the test.

The organization of the paper is as follows: Section 2 deals with the materials and methods of the proposed research work. It describes the waveguide method for dielectric properties measurement of edible flour samples. Section 3 deliberates the results and discussions of the experiments; it relates the attenuation constant, fibre and absorption of edible flours. Section 4 presents the conclusion of the research work.

## 2. Literature Review

Food fibre plays an important role in human health and well-being. Food rich in fibre includes fruits, vegetables, beans, nuts and dairy products. Among these resources, grains are the fibre key factor to humans, as they are consumed in daily meals. In most parts of Southeast Asia regions, rice is the staple grain, and wheat, commonly in the form of bread, is the staple daily consumption of the rest of the world. Millets, on the other hand, despite known as containing significant nutrients, are less commonly consumed [11]. Daily consumptions of these grains are, therefore, among influences that contribute to human nutrition, which nourish the wellness of our body.

The nutrients in grain provide balanced fibre, which is termed by food technologists as dietary fibre [12,13,14,15,16,17,18]. These grains are knowingly, or perhaps unknowingly to the majority, critical to the human immune system. For this reason, in order to educate and motivate the global population, the World Health Organization (WHO) and other non-profitable organizations provide basic guidelines and awareness of how to follow a healthy diet [19]. Subsequently, in the present day every food that is consumed is labelled with nutritional information on its packaging contents, as an effort to keep the consumer informed on the food intake. Human survival is crucial to the growth of the immune cell in human physics [20]. There is a risk of disease to humans when they consume low-nutritious meals [20]. Since these fibres are non-digestive to the human digestive system [17,18,19,20,21,22], various severe chronic diseases, such as gastrointestinal tract [23,24], diabetes [25] and colorectal neoplasia (rectum colon) cancer [26], may cause destruction to the digestive system. Contrarily, however, literature has correspondingly reported the benefits and advantages of fibre in reducing cancer and cardiovascular disease and the risk of diabetes [15].

Nevertheless, it is scientifically proven that fibres are undoubtedly very rich in grains [27]. Whole grains benefit to healthy diet was recently reviewed by [27], addressing its advantages. Regrettably, according to this grains review [27] and consumers survey [28] and recent surveys [29,30], they reported and observed that intakes of whole grains is low [27,28,29,30]. Refined grains are more preferable and the perception of whole grains is not fully appreciated. Rice, likewise, is the main food in many Asian countries; white rice intakes are cultured and habitual. Intake of brown rice, which is scientifically proven to be more nutritious [27] than its wheat counterpart, were low. Additionally, a recent study by Watanabe et. al [31] found that countries with rice as an eating habit have more resistance to the COVID-19 virus than countries with bread as their staple food. Nevertheless, as grain products reach consumers in the form of edible and refined food, the percentage of fibre still exists. Carbohydrates, for example, are usually perceived as only being left with a source of energy without fibre when grains are refined [3]. However, the dietary fibre research findings proved that fibre carbohydrates still contain fibre in the grains of refined form [32,33]. Nonetheless, although vital fibre is lacking in diets and beneficial for the bodies, consensus has to be ensured, especially in today’s world and market, when varieties of worldwide food can be reached and consumed globally. It is, therefore, necessary to identify and measure the fibre contents of these foods.

Identification of food composition is essential not only to ensure its safety but also to publicise the contents. The biochemical method is the typical technique to identify the fibre content in food [33,34,35,36]. The samples of food are tested in laboratories with common chemical instruments to test the food composition with chemical reactions. The tested samples are then quantified under microscopes with certain conventional chemical techniques to recognize the food composition [36]. These methods are standardized to guide particularly the food industries to follow the national, as well as international, measurement standard, in order to ensure quality food reaches consumers all over the world. The amount of fibre contents in food is standardized but was not until early 1990s when the United States of America Foods and Drugs Administration (FDA) regulated the methodology in their standards [36,37]. Currently, the Association of Official Agricultural Chemists (AOAC) is the accredited authority used by government agencies to certify food composition standards [38].

Unlike biochemical tests, common global standard fibre identification using microwave dielectric is seldom available. However, the American Society of Agricultural and Biological Engineers (ASABE) has approved and adopted a model [39] proposed by Nelson in their publication on dielectric metrology for cereal grains in 2012 [40]. The method adopted is the free-space transmission technique. This technique was chosen, as the evidence composed is comparative. The entire volume of the sample is sufficiently interacting with the occurrence of the electric field. This instance of electricity helps the propagation of the electromagnetic wave. Consequently, the measured dielectric properties representatively present the sample material. Other advantages of this measurement technique consist of non-physical contact with materials and its nominal sample preparation [40]. Perhaps, Stuart O Nelson is the most notable figure in this field as he and his associates have explored this dielectric measurement since the late 1980s. The long period of research has gained them useful amounts of data in developing microwave dielectric sensing equipment, which applies particularly for grain dielectric measurement.

The essentiality of foods microwave permittivity [41] or dielectric properties in moisture contents [41,42,43] drying system design and control has attracted interest among scientists and researchers in this field. Microwave is an electromagnetic wave that functions in a wide range of frequencies ranging from 300 MHz to 300 GHz [44,45]. The usage was initially meant for radar in World War II [46]. The microwave oven that we see today was popularized in the 1960s and was earlier invented by Percy Spencer in 1946 [46]. The oven operates at a frequency of 2.45 GHz [22,45]. The advantage of short cooking time conveniently suits the 24/7 fast rapid lifestyles of humans today. The oven has become a necessary appliance in the common modern home. It is not only used to thaw frozen food but also used for cooking. Its potential largely benefitted from the waveguide that permits electricity to function through molecule polarization for the heating processes [45,46,47,48].

These electromagnetic metrological traits correspondingly benefit for the purpose of measurement, especially small particles, such as grains, which did not require heating or thermal energy in the process. One of the main reasons for the growing interest factor in using microwave as the measurement is because the dielectric exists in foods. As mentioned in the Section 1, using dielectric technique has potential in assessing the food quality. Since these properties contain core information, it may correspondingly be useful for the food assessment. Studies on microwave waveguide methodology to determine the dielectric properties in cereals was reported in [3]. Recent review publications on microwave dielectric properties on food processing and various dielectric properties of food have reported its advantages and its applications to several types of food [45,49,50]. Guzik et al. [49] focus their review on the benefits of microwave in the food processing industry (drying, sterilization, pasteurization, thawing, etc.). Teseme et al. [50] reviewed mainly on food materials (cereals, bakery products, fruits and vegetables, etc.). Abo Bakar [45] highlighted microwave heating, cooking, baking, drying, blanching, pasteurization and sterilization. Another quite recent review by Tiras et al. [51] reported the dielectric properties in various forms of food (meat, eggs, dairy products, flours, etc.). Moisture contents of barley and corn, sorghum and wheat were reported by [52,53]. In addition, [53] reported microwave absorption in soybeans, canola, shelled and pod peanuts. Rice flour moisture contents by microwave absorption was reported by [54]. Many other publications, e.g., Zhang et al. [55] solely reported on the pecan nut properties of dielectric.

The dielectric properties of the food attenuate the microwave frequencies. Microwave attenuation refers to the absorption of the microwave radiation energy [54]. It is basically the input and output ratio of the electromagnetic energy produced. The attenuation measurement requires system architecture or models to quantify the data throughout the experimentation. The techniques of dielectric properties measurement started in 1954 [40]. Ever since then, sensing fibre content by microwave attenuation has been successfully reported in recent publications [56]. However, in terms of model that were used to identify the fibre content in food composition by microwave technique, minimal literature has been published or reported. The early measurement model can be traced back and credited to [57,58]. The most recent studies made available in the literature are reported by Kalandarov et al. [59]. A model of moisture microwave meter was developed making use of an automatic moisture meter they developed and patented earlier in 2010 [59]. Thus, their recent study focused on the model, which was then used to experiment on cotton seed and wheat grains [59] to observe the microwave attenuation. Another recent valid related study was presented by Li et al. [60]. Similar to Nelson [61], the proposed model, however, modified the antenna and used a constantly moving antenna instead, for uniform accuracy of microwave radiation to the sample measured. The antenna moved in the horizontal direction so as to measure the sample in the container. The measurement used in this article, however, used the two-point method [62]. This methodology was adopted, since the dielectric properties measured in this article is in powdered form. The research article proposed by Darwish et al. [63] and Gabor et al. [64] shows fibre content analysis using various methods.

## 3. Materials and Methods

Once microwave energy is absorbed, polar molecules, such as water molecules inside the agricultural products will rotate according to the microwave. The water molecule is a “dipole” with one positively charged end and one negatively charged end. Similar to the action of a magnet, these “dipoles” will orient themselves when they are subject to microwaving. The rotation of water molecules would generate heat in the substance. This phenomenon is normally called dipolar interaction. Another phenomenon is called ionic interaction, due to ionic compounds (i.e., dissolved salts) in agricultural products that can also be accelerated by the microwave and colliding with molecules that produce heat into the products. Hence the composition of the agricultural products will be affected like when it is heated up inside the microwave oven. Agricultural products with higher moisture content will be heated up faster because of the dipole interaction. As the concentration of ions (e.g., dissolved salts) increases, the rate of heating also increases because of the ionic interaction with microwaves. Even though oil molecules are much less than water molecules and are non-ionic, agricultural products with high oil content have a fast-heating rate because the specific heat of the oil is less than half that of water.

Permittivity is the measure of resistance that is encountered when forming an electric field in a medium. The dielectric constant is the ability of a material to store energy in the electric field in the material. The loss factor is the measure of energy absorption of the material. The formula for permittivity [1] is given below in Equation (1)

(1) ε =έ− jἔ

where ε = relative complex permittivity, έ = relative real permittivity (dielectric constant), ἔ = dielectric loss factor, j = imaginary unit.

The dielectric constant [61] is given as

(2) $$έ={\left(\frac{\beta }{\beta_{o}}\right)}^{2}$$

Similarly, the loss factor [61] is given as

(3) $$ἔ=2\alpha \left(\frac{\beta }{\beta_{o}}\right)$$

where α is attenuation constant and β is phase constant.

Attenuation is the gradual loss in intensity of any kind of flux through a medium. The value of attenuation differs for the different flours depending on the nature of the substance. The attenuation constant is calculated from the microwave waveguide experiment as given in Equation (4).

(4) $$\alpha =20log\left(\frac{V_{1}}{V_{2}}\right)$$

where $V_{1}$ is forward voltage without sample and $V_{2}$ is forward voltage with the sample.

The phase constant is given as

(5) $$\beta =\frac{2\pi }{\lambda_{g}}$$

The dielectric constant and loss factor can also be determined by the other parameters of the waveguide; guided, free space and cut-off wavelength.

Dielectric constant (έ) [62] is given as

(6) $$έ={\left(\frac{\lambda_{o}}{\lambda_{c}}\right)}^{2}+{\left(\frac{\lambda_{o}}{\lambda_{c}}\right)}^{2}\left(1-{\left(\frac{\alpha }{\beta }\right)}^{2}\right)$$

where $\lambda_{o}$ is the free-space wavelength, $\lambda_{c}$ is the cut-off wavelength

The dielectric loss factor [62] is given in the following Equation (7).

(7) $$ἔ=2\left(\frac{\lambda_{o}}{\lambda_{c}}\right)\left(\frac{\alpha }{\beta }\right)$$

And also guided wavelength is given in the following Equation (8).

(8) $$\lambda_{g}=2\left(d_{1}-d_{2}\right)$$

where $d_{1}$ is the point of first minima in vernier scale reading in the microwave slotted line section, $d_{2}$ is the point of second minima in vernier scale reading in the microwave slotted line section.

Free space wavelength [62] can be calculated from the following Equation (9).

(9) $$\frac{1}{\lambda_{o}}=\sqrt{{\left(\frac{1}{\lambda_{g}}\right)}^{2}{\left(\frac{1}{2a}\right)}^{2}}$$

where a is the dimension of the rectangular waveguide.

The cut-off wavelength [62] is given in the following Equation (10).

(10) $$\lambda_{c}=2a$$

where a is 2.286 × 10^2^ m.

The complex dielectric constant [65] is given in Equation (11)

(11) $$C∠-\psi =\frac{1}{j\beta I_{e}}\frac{1-\left|\Gamma \right|}{1+\left|\Gamma \right|}\frac{e^{j\psi }}{e^{j\psi }}=\frac{tanX∠\theta }{X∠\theta }$$

where C is the magnitude of the complex quantity, ψ is the phase of the complex quantity, the term X∠θ  is the solution to the complex dielectric constant. *I_ε_* is length position in slotted line section.

The admittance ($Y_{\varepsilon }$) of the material of the sample [65] is given in Equation (12)

(12) $$Y_{\varepsilon }=\left[\frac{X}{\beta I_{\varepsilon }}\right]∠2\left(\theta -90^{{}^{\circ}}\right)=G_{\varepsilon }+jS_{\varepsilon }$$

where $G_{\varepsilon }$ is the conductance of the sample and $S_{\varepsilon }$ is the susceptance of the sample

The values of $G_{\varepsilon }$ and $S_{\varepsilon }$ are obtained by separating this equation into real and imaginary parts, which provide the values of έ and ἔ [65] in the following form

(13) $$έ=\frac{G_{E}+{\left(\frac{\lambda_{g}}{2a}\right)}^{2}}{1+{\left(\frac{\lambda_{g}}{2a}\right)}^{2}}$$

(14) $$ἔ=\frac{-s_{\varepsilon }}{1+{\left(\frac{\lambda_{g}}{2a}\right)}^{2}}$$

The most general description for electromagnetic purposes of a given homogenous material is given by complex permittivity (dielectric constant) together with complex magnetic permittivity.

The dielectric constant of the material [65] is given in Equation (15)

(15) $$έ={\left(\frac{\lambda_{0}}{2\pi }\right)}^{2}{\left(\frac{2\pi }{\lambda_{c}}\right)}^{2}-\left(\alpha^{2}-\beta^{2}\right)$$

The dielectric loss factor of the material [65] is given in Equation (16)

(16) $$ἔ={\left(\frac{\lambda_{0}}{2\Pi }\right)}^{2}\left(2\alpha \beta \right)$$

where $\lambda_{0}$ is free space wavelength and $\lambda_{c}$ is the cutoff wavelength.

The penetration depth of the material [65] is given in Equation (17)

(17) $$d_{p}=\frac{\lambda_{0\sqrt{έ}}}{2\pi ἔ}$$

**Figure 1:**
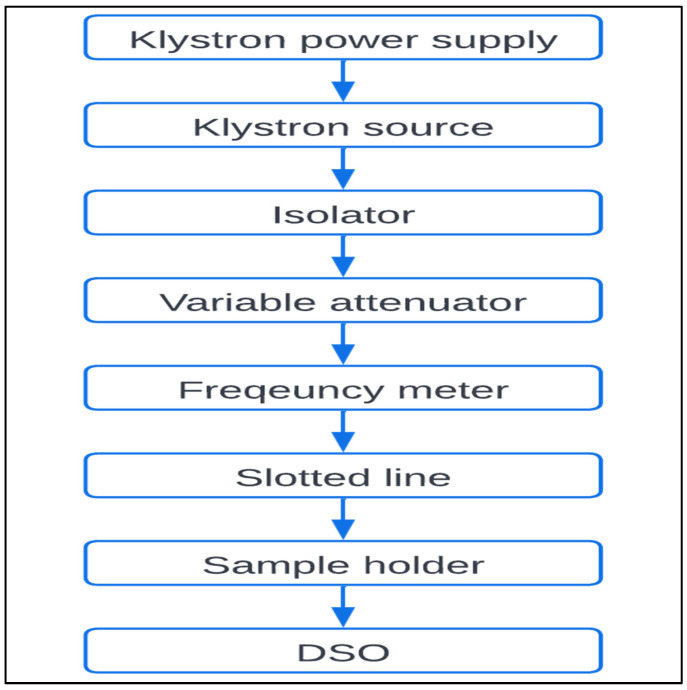
Block diagram for waveguide method.

To measure the electrical properties of cereals, the microwave waveguide method approach is used. The generalized process of the method is shown in Figure 1. The samples are placed in the sample testing waveguide and microwave is passed through it. The klystron source produces microwave energy. The sample absorbs microwave energy, based on the nature of samples, according to their material properties, and thus the attenuated output voltage is observed to determine the attenuation properties of the samples.

Two-point methods are used to measure dielectric constant, dielectric loss factor and attenuation of edible flours [3].

The waveguide is filled with the powdered samples and the samples are placed into the waveguide at a constant weight of 150 g for repeated observation for more samples. When the microwave is passed through the sample, the sample absorbs a certain amount of microwave energy. This absorption attenuates the input microwave, and hence accordingly the output microwave signal strength gets reduced. Now the attenuated microwave at the output stage is measured. From the measured output voltage from the digital storage oscilloscope (DSO), which stores and helps to analyse the microwave signals, the amount of absorption can be determined using the above equations. The quantity of absorption depends on the number of cereals present in the samples and the nature of the samples inside the waveguide. We follow the same procedure to find the attenuation for cereals. The attenuation is calculated for the powdered and filtered samples.

## 4. Results and Discussions

**Figure 2:**
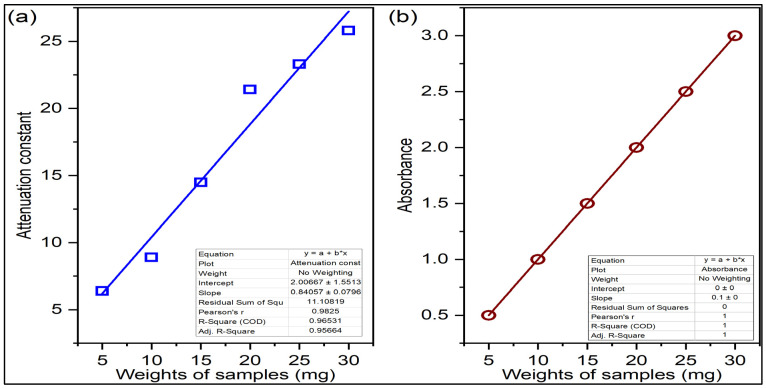
(**a**) Attenuation constant and (**b**) absorbance vs. weight of sample in mg. The blue colour line indicates attenuation constant and the brown colour line indicates absorbance. Linear equations for attenuation constant by the waveguide method from the graph as y=0.84057x+2.0067, where the slope of the graph is m=0.84057. The squares and circles represent the data for attenuation constant and absorbance respectively.

Based on the experimental results, the following inferences are made from this experimental investigation. In this work, different kinds of edible flour, such as wheat, rice and millets, are considered to determine their quality. The wheat, rice and millet products are rich in terms of carbohydrates, which is the biomolecule present in wheat, rice and millets. These flours are treated with a light heating process to remove the moisture contents in the samples before placing them into the testing waveguide or dielectric cell under test. The moisture content present in the test samples will introduce additional absorption to the microwave while the microwaves are passed through the waveguide with samples. The qualities of the wheat, rice and millet flours are now mainly disturbed by the lack of fibre in the flours. The food processing industries remove the fibre contents of the flour, which would also affect the nutritional value of the food. Thus, this work is mainly concentrated on the presence of fibre content in the edible flours by estimating the attenuation to the microwaves by the edible flours. The fibre content is a vital nutrient in the edible flours and the concentration of fibre contents in the edible flour can be related to the attenuation of the flours to the microwave energy. It is observed that the concentration of fibre content in the sample flour reduces the absorption of microwave energy and the attenuation constant of the sample gets reduced. The value of the attenuation constant is more for less concentration of fibre content in the flour. It is noted in Table 1 that the value of the microwave attenuation constant is increasing while the amount of edible flour inside the waveguide is increasing. It is observed that the higher the sample amount, the greater the attenuation constant. At first, the experiment is tested for its linearity in attenuation constant with an increase in the sample amount that is given in Figure 2 below.

Estimation of fibre in wheat flour: This microwave waveguide approach is also used to reveal the presence of fibre in the edible flours. maida flour is fibre-free edible flour, and it is considered the reference for determining the presence of fibre in the flour. At the same time as comparing the attenuation constant of maida for a given amount of weight, the reduction in attenuation constant for other edible flour from that of maida for the same amount of weight indicates the presence of fibre in it. Thus, the higher the fibre content in the flour, the lesser the value of the attenuation constant.

From the above Table 2, it is inferred that among the seven types of wheat, the Punjab wheat has the least attenuation constant among the others, since the fibre content is the highest for Punjab wheat. The attenuation constant for Punjab wheat, Samba wheat, local wheat, Ration wheat, Aashirvadh^TM^ wheat, Pillsbury^TM^ wheat and maida flour is 16.6, 17.69, 17.98, 19.27, 22.13, 23.71, 29.17, respectively. As maida flour has no fibre content in it, its absorption of microwave energy is more than that of other flours used in this observation, which is also proved by the experiments through its greater value of attenuation constant.

**Figure 3:**
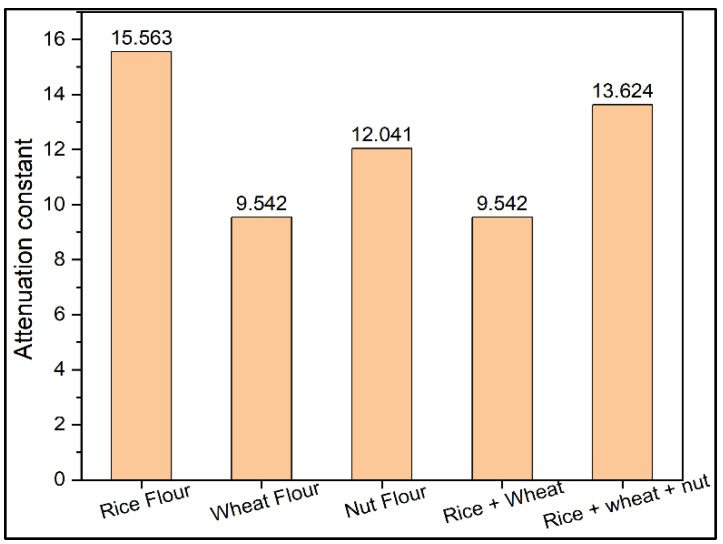
Attenuation of rice flour, wheat flour, nut flour and their mixtures.

**Figure 4:**
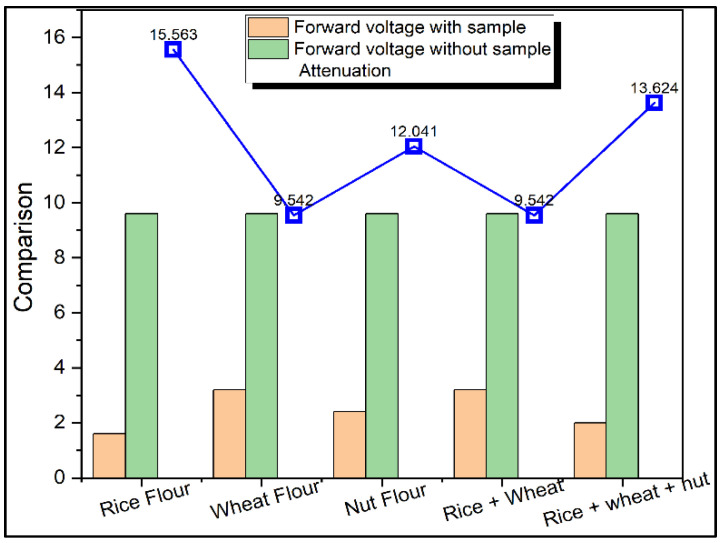
Comparison of input voltage, output voltage and attenuation for rice flour, wheat flour, nut flour and their mixtures.

From Figure 3, wheat flour has low attenuation constant than rice flour, nut flour and their mixtures because wheat has low absorption due to having more fibre content in it. Figure 4 shows the comparison of input voltage, output voltage and attenuation for rice flour, wheat flour, and nut flour and their mixtures. Wheat flour has the lowest attenuation constant among rice flour, nut flour and their mixtures due to low absorption in wheat flour by the presence of fibre content.

**Figure 5:**
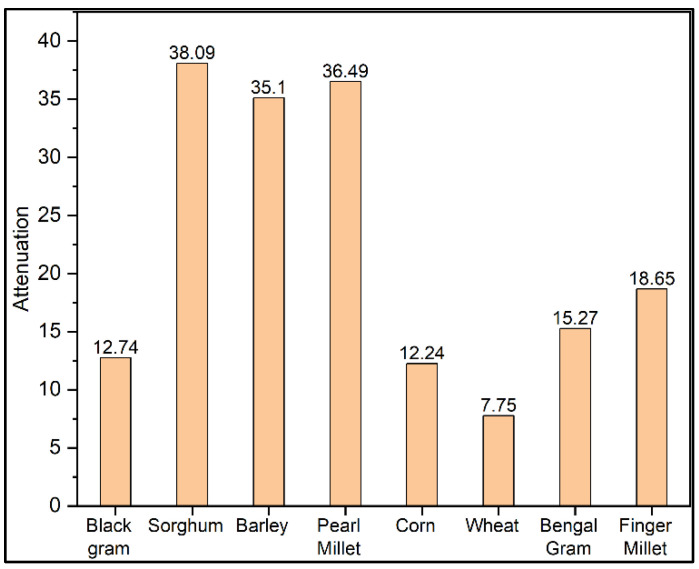
Attenuation constant for various cereals.

**Figure 6:**
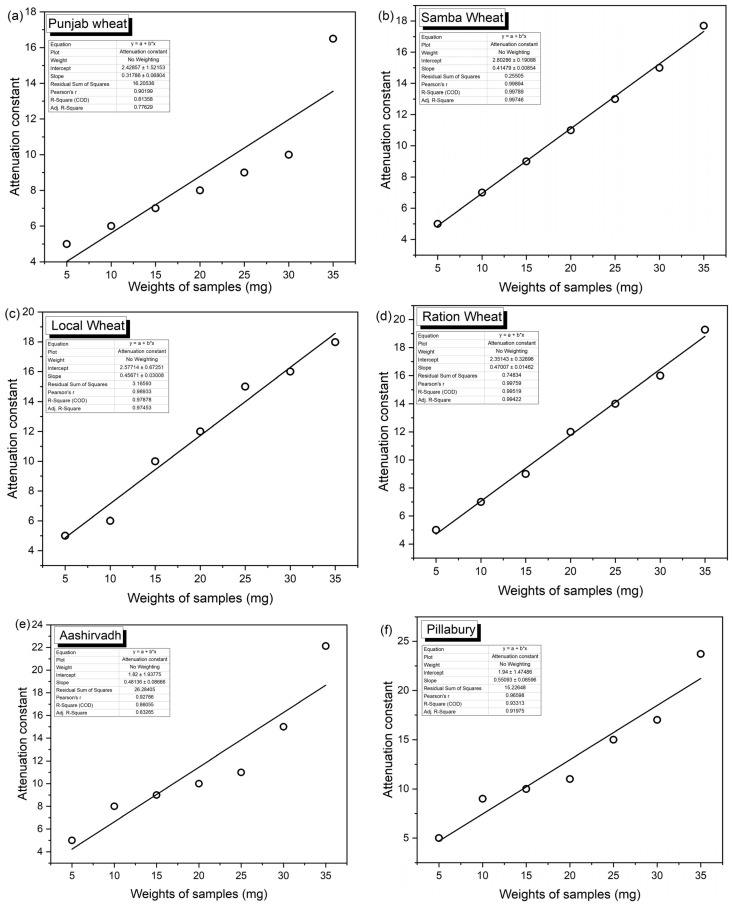

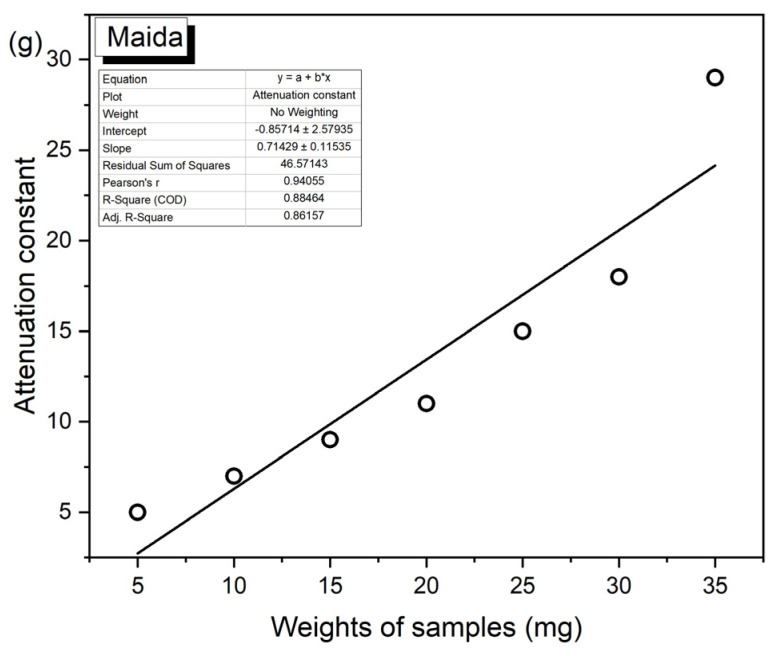
Attenuation constant for various cereals versus weight of samples (**a**) Punjab wheat (**b**) Samba wheat (**c**) Local wheat (**d**) Ration wheat (**e**) Aashirvadh^TM^ (**f**) Pillabury^TM^ (**g**) Maida.

When wheat flour is adulterated with low fibrous rice flour and/or nut flour, the attenuation gets increased, and hence it shows less quantity of fibre in the mixture. Figure 5 shows the attenuation constant for black gram, sorghum, barley, pearl millet, corn, wheat, Bengal gram and finger millet. The least attenuation constant is for wheat. Figure 6 shows the comparison of various wheat flours attenuation constant for the weight of samples in which Punjab flour has the least attenuation constant among other wheat flours, and hence its fibre content is more than that of other wheat flours.

From the above experimentation, it is observed that the rich fibre content of edible flours makes a decrease in absorption of microwave energy, which leads to reducing the attenuation constant. This is proved and witnessed from the experiments conducted for these edible flours using the microwave workbench in the laboratory.

Initial conditions:

Beam voltage = 230 V Repeller voltage = 240 V Beam current = 21 mA

Dip Frequency = 9.8 GHz without sample = 320 mv

**Figure 7:**
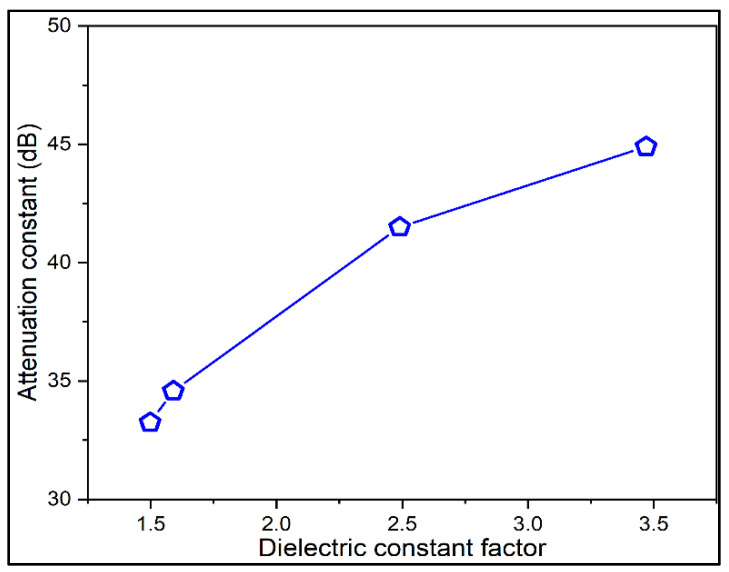
Attenuation versus dielectric constant of wheat flours.

Table 3 illustrates the relationship between various electrical parameters, such as attenuation constant, dielectric constant, permittivity and loss factor. All these parameters have the same tendency in their variation throughout the investigation. Samba wheat has the least value for the parameters listed in the table. The relation between the dielectric constant and attenuation constant is also indicated in Figure 7 to validate the uniform variations among them. Hence, estimating the attenuation constant alone for all cases of edible flour is sufficient to justify the presence of fibre content in the substances. Moreover, the attenuation constant of maida flour is the highest one that indicates it is that edible flour has less fibre content which is obvious to everyone in the society. Thus, the reliability of this investigation and the methodology is proven by this research. To validate the results obtained in this work, standard deviation (SD, also represented by the Greek letter sigma *σ*) is also measured for multiple readings for the same kind of samples at various time intervals that are used to quantify the amount of variation or dispersion in a set of data values.

Standard deviation is given by

(18) $$\sigma =\sqrt{\left(E\;\left[{\left(X-\mu \right)}^{2}\;\right]\right)\;}$$

Table 4 lists the average of standard deviation measurements that indicates the deviation of the successive readings of attenuation constants, which is noted as very minimal for all cases of experiments. Thus, the standard deviation analysis also reveals the reliability of this investigation of edible flours. The standard deviation observation of the experimental results of the attenuation constant is presented in Table 4. Maida provides no deviation in normalized attenuation because it is almost fibre-free edible flour.

## 5. Conclusions

An alternate approach for the identification of fibre content in edible flour is presented. The dielectric properties of cereals in powder form are determined by using the microwave waveguide method. The identification of fibre content in edible flour by the biochemical method is a challenging task. The proposed method supports for finding fibre content of edible flours, such as wheat, rice and millets. The dielectric cell having edible flour inside it acts as a microwave waveguide. The microwave absorption by the samples inside the waveguide reflects the richness of fibre content of the sample. The presence of fibre in the sample is mapped by estimating the attenuation constant of the sample under test. The experimental research shows that the richness of the fibre content reduces the absorption of microwave energy, and hence attenuation constant gets reduced. Thus, it is possible to identify the fibre quality of edible flour by this proposed approach. The reliability of the experimental results is validated in this work by suitable quantitative parameters, such as the standard deviation of the experimental results and physical verification, is also responsible for the correctness of the results. Various kinds of wheat flours and rice flours are investigated for observing fibre content in the flour. In many of the cases, wheat flour shows the highest fibre content and maida has the lowest fibre content. This approach will be the best alternative to the existing biochemical tests in case of fibre identification of edible flours that will support health care and the food industry.

## Figures and Tables

**Table 1:** Attenuation constant for wheat flours for different weights of samples.

	Without Sample	5 mg	10 mg	15 mg	20 mg	25 mg	30 mg
V_pp (Peak-Peak Output Voltage)_	2.36 v	1.12 v	840 mv	440 mv	200 mv	160 mv	120 mv
Attenuation constant		6.47	8.97	14.59	21.44	23.38	25.87

**Table 2:** Attenuation Constant of Different Variety of Wheat Flours.

Samples (20 g)	Peak-Peak Voltage (v)	Power (mW)	Attenuation Constant (dB)
Punjab Wheat	2.72	0.378	16.6
Samba wheat	2.4	0.28	17.69
Local wheat	2.32	0.34	17.98
Ration wheat	2	0.14	19.27
Aashirvadh^TM^ wheat	1.44	0.13	22.13
Pillsbury^TM^ wheat	1.2	0.12	23.71
Maida	0.56	0.04	29.17

**Table 3:** Electrical parameters of different varieties of wheat flours.

Samples (20 g)	Peak–Peak Voltage (mv)	Power (μW)	Attenuation Constant (dB)	Dielectric Constant	Loss Factor	Permittivity
Samba	6.96	146.16	33.25	1.498	112.53	112.54
Ration	5.97	125.37	34.58	1.59	135.10	135.11
Aashirvadh^TM^	2.72	57.12	41.50	2.49	375.30	375.31
Maida	1.80	37.8	44.9	3.47	662.6	662.61

**Table 4:** Attenuation constant of different varieties of wheat flours.

Standard Deviation	Aashirvadh^TM^	Ration Wheat	Samba	Maida
Sample 1	0.36	0.77	0.32	0
Sample 2	0.36	0.17	0.32	0
Sample 3	0.76	0.23	0.72	0
Sample 4	0.36	0.17	0.52	0
Sample 5	0.24	0.77	0.32	0
Sample 6	1.24	0.17	0.32	0
Sample 7	0.36	0.17	0.52	0
Sample 8	0.76	0.77	0.52	0
Sample 9	0.36	0.23	0.32	0
Sample 10	0.36	0.17	0.52	0

## Data Availability

The data presented in this study are available through email upon request to the corresponding author.
